# Is interstitial 8p23 microdeletion responsible of 46,XY gonadal dysgenesis? One case report from birth to puberty

**DOI:** 10.1002/mgg3.558

**Published:** 2019-01-28

**Authors:** Kathy Wagner‐Mahler, Jean‐Yves Kurzenne, Frederique Gastaud, Marie Hoflack, Patricia Panaia Ferrari, Etienne Berard, Fabienne Giuliano, Houda Karmous‐Benailly, Pamela Moceri, Celine Jouannelle, Marine Bourcier, Elise Robart, Yves Morel

**Affiliations:** ^1^ Département de Pédiatrie Centre Hospitalier de Nice Nice France; ^2^ Hôpitaux Pédiatriques de Nice CHU Lenval Nice France; ^3^ Département de Biochimie Centre Hospitalier de Nice Nice France; ^4^ Département de Génétique Centre Hospitalier de Nice Nice France; ^5^ Département de Cardiologie Centre Hospitalier de Nice Nice France; ^6^ Centre Hospitalier Universitaire de Lyon – HCL GH Est, Centre de Biologie et Pathologie Est Bron France

**Keywords:** 46,XY disorders of sexual differentiation, 8p23 microdeletion, *GATA4* gene, gonadal dysgenesis, undescended testis

## Abstract

**Background:**

Chromosome 8p deletions are associated with a variety of conditions, including cardiac abnormalities, mental, behavioral problems with variable morphotype and genitourinary anomalies in boys.

**Methods:**

We describe the follow‐up over almost 15 years of a boy who initially presented with perineal hypospadias with a micropenis and cryptorchidism with 46,XY DSD.

**Results:**

Imaging, pathology, and hormonal exploration suggested gonadal dysgenesis. Further genetic studies were deemed necessary during follow‐up. The child's further development recommended further genetic analyses. High‐resolution analysis showed an interstitial deletion on the short arm of a chromosome 8: 46,XY,del(8)(p23.1p23.1). We reviewed the literature and found 102 cases including 54 boys: 62.7% had mental problems, 50.9% a dysmorphic disorder, 55.9% cardiac anomalies, and 46.3% of the boys had genitourinary anomalies. Our patient's genital abnormalities can be explained by the haploinsufficiency of the genes, such as *GATA4* (OMIM 600576) that are included in the deleted area.

**Conclusion:**

This case of severe 46,XY DSD raises the question of the role played by 8p23 microdeletion in gonadal dysgenesis. Clinicians are encouraged to look for this anomaly on chromosome 8 in cases of unexplained gonadal dysgenesis even when few signs suggestive of this anomaly are present.

## 1 INTRODUCTION

The etiological search is often negative in those cases of 46,XY disorders of sexual differentiation (DSD) without familial history (Ahmed, Khwaja, & Hughes, 2000; Ahmed, Cheng, et al., 2000; Morel, Rey, & Teinturier, 2002).

We report the case of a boy born with insufficient masculinization of the external genitalia, a 46,XY DSD and other associated signs. There was no familial history, and the prenatal standard karyotype was considered normal. However, the child's morphotype (microcephaly and facial abnormalities) and psychomotor development (delayed walk and language) lead us to seek for other genetic abnormalities involved in hypo‐masculinization. Only a high‐resolution karyotype yielded to the diagnosis of an interstitial 8p23 deletion.

This deletion is often reported in patients with delayed psychomotor development and cardiac anomalies but classically not in patients with primary diagnosed genitourinary malformations which, the literature shows, are reported in almost a boy in two.

## 2 CLINICAL DESCRIPTION AND PATIENT MANAGEMENT

### 2.1 From birth to age 2

**Figure 1:**
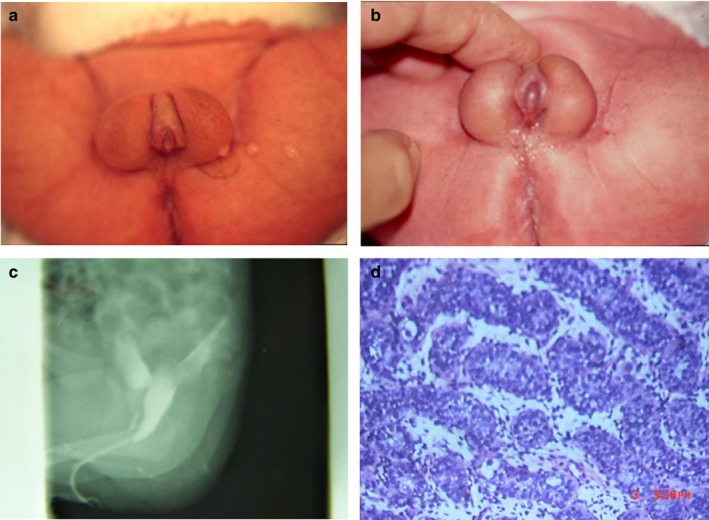
Genitalia abnormalities (a) & (b) External genitalia at 3 weeks. (c) Genitocystography. (d) Testicular biopsy

This male patient was born after 37 weeks of amenorrhea by cesarean delivery of healthy and no consanguineous parents (Figure 1). His mother was 40 years old. A prenatal amniocentesis motivated by the mother's age showed a normal 46,XY karyotype.

The external genitalia abnormalities discovered at birth consisted in a perineal hypospadias with a micropenis and cryptorchism. He weighed 2,630 g, his height was 47 cm, and his cranial circumference was 33 cm (−1 *SD* harmonious). He showed unspecific face characteristics: big ears with low implantation, and prominent nose. We observed a single crease in the palm of the right hand and a “café au lait” spot of 2.5 cm diameter on the inside of the left leg. There was no other malformation. The phenotype at birth suggested no specific syndrome.

At 3 weeks of life, the child was assessed by the medico‐surgical team. The external genitalia were described as perineal hypospadias and micropenis of 15 mm with chordee. The scrotum was moderately differentiated with a slight transposition, and the testis was palpable in a high position, but could not be descended.

Medical imaging showed the presence of a retrovesical cavity, and the size of the testicles could be precisely measured: 13 × 6 mm for the left testis, right 14 × 6 mm for the right testis.

The genito‐cystography showed a posterior vaginal cavity with a low positioned opening of the vagina in the urethra. There was no urinary ducts abnormality. The kidneys were small (−2*SD* for the right kidney, −1*SD* for the left kidney) but their echogenicity was normal. We searched several times for microalbuminuria with negative results.

The echocardiography was normal. No other anomaly was found.

At birth, the anatomical appearance was more in favor of a male sex, and the child was declared as a boy. For this reason, no sexual reorientation has ever been considered during follow‐up.

The first‐intention treatment was hormonal: Two androgen cures with diagnostic and therapeutic intent were performed using 100 mg/m² testosterone enanthate, four intramuscular injections at 2 weeks interval. The first, at one month of age, resulted in a lengthening of the penis from 15 to 25 mm, followed by a second cure at age 3.5 months, with satisfactory results, the length of the unbent penis reaching 40 mm at the end of treatment. This treatment showed a good tissular receptivity to androgens.

The next step was the surgical treatment at 11 months. A masculinizing genitoplasty was undertaken: Duckett's pedicle tube urethroplasty (4 cm flap) with Duplay procedure and bilateral orchidopexy. The posterior vaginal cavity was left in place. With the maternal consent, a testicle biopsy was performed for anatomopathologic examination.

The testis had a well‐developed albuginea in half of its surrounding that became thinner in the other part with tubule inclusions. The tubules were densely packed in the normal area but less numerous and branched in the abnormal portion, leading to the diagnosis of dysgenetic testis. The search for SRY (sex‐determining region of Y chromosome, OMIM 480000) was positive in Sertoli cell nuclei while WT1 (Wilms tumor 1, OMIM 194070) was negative. AMH (anti‐Mullerian hormone) in Sertoli cell positivity was lower than normal for the age, and AMH‐R (AMH‐receptor) was abnormally located inside their nuclei. Germ cells are present inside tubules but in reduced number (germ cell hypoplasia).

### 2.2 From age 2 to puberty

A second surgical intervention was necessary at age 2 to treat a fistula due to a failure of the distal half of the Duckett's tubule. The child had urinary leaks and frequent urinary infections. Bladder control was long to achieve, and the child was finally dry at 3 years and 10 months with slight urinary leaks persisting after mictions. Urinary infections became more and more frequent, without fever but with increasingly resistant germs. Intravenous urography and cystography showed that the urethra reconstruction was probably not the cause of the problems. The kidneys were small (right and left −2 *SD*).

At age 5, the patient had evolved harmoniously with a weight and height at +1 *SD* and, although it had stayed anatomically stable until then, it was decided to remove under coelioscopy the residual vaginal cavity. The surgical ablation of these mullerian residues with preservation of the vas deferens solved the infectious problem.

With regard to the delayed psychomotor development, we observed that the cranial circumference stopped growing at age 18 months yielding to a microcephaly (−4 *SD*). The child was able to sit at 9 months and to walk at 2 years. Language acquisition was strongly delayed. The patient presented behavioral troubles with major psychomotor instability and concentration problems. No objective neurological symptom was identified. No seizure was ever observed. A brain MRI performed at 3 years and 2 months was considered normal. School attendance in kindergarten was possible with specialized help, with a psychomotor and psychological follow‐up.

At age 11, the child started a spontaneous puberty with asymmetric development of the testicles (better testis development left compared to right). At the age of 13.5 years, a stagnation of the sexual development was noticed: The penis measured 70 mm × 25 mm, testicles volume was 10 ml, P4, A3 hairiness and appearance of moderate gynecomastia. Replacement therapy with testosterone heptylate was introduced. At the same period, the patient was referred to the cardiology department to investigate an abnormal heart murmur. Transthoracic echocardiography and cardiac magnetic resonance were consistent with the diagnosis of left ventricular noncompaction (hypertrabeculated left ventricle, preserved ejection fraction, moderate functional mitral regurgitation).

At 14 years 8 months, the growth curve bended: 172 cm size (target height 179.5 cm) with a weight of 53.5 kg. He still needs special help in a normal school setting. Behavioral problems were treated with risperidone and psychotherapy.

In conclusion, we report the case of a child presenting a 46,XY gonadal dysgenesis, with microcephaly but no characterized facial dysmorphy, transverse palmar crease, slight psychomotor impairment, and behavioral who also developed after puberty a left ventricular noncompaction.

## 3 DIAGNOSTIC HORMONAL INVESTIGATIONS METHODS

### 3.1 Baseline hormonal investigations

Serum was collected in gel separator tubes (BD Vacutainer system SSTII, Becton‐Dickinson), and cells were separated from serum by centrifugation (Table 1). The samples were stored at −20°C before testing. All tests were performed twice for control reasons with the same assay.

**Table 1:** Hormonal investigations results

Baseline hormonal investigations	At 1 month	At 7 months	At 10 months	At 14 months	At 24 months	At 6 years	At 11.5 years	At 13.5 years
Testosterone (nmol/L)	4.16	2.08	0.67	0.17	<0.3	<0.3	3.1	10
DHT (nmol/L)	4.26	<0.21						
Inhibine B (pg/ml)	52	<15		29		<10	55	69.6
FSH (U/L)	7.9	<1.5	1.8			<1	12.8	27
LH (U/L)	19	1	1			<1	3	8.2

Hormonal stimulation tests LH RH test at 10 months	0 min	10 min	20 min	40 min	60 min	120 min		
FSH (U/L)	1.8	9	15.8	18.3	**20.6**	14.1		
LH (U/L)	<1	4.9	8.2	**9.3**	7.6	3.7		
Testosterone (nmol/L)	0.69					<0.34		
r‐HCG test at 14 months	J0	J3	J7					
Testosterone (nmol/L)	0.19	3.2	3.9					

Notes.

Peak values are in bold.

Testosterone, FSH, and LH dosages were performed on a ADVIA Centaur automaton (Bayer Diagnostic) using a competitive immunoassay using direct chemiluminescent technology. Delta 4 androstenedione was measured with RIA DSL‐3800 Active Androstenedione (Diagnostic System Laboratories, Webster, TX) which follows the basic principle of radioimmunoassay. 17OHP was measured by the RAI technique (Kit DSL‐5000 Active 17 OHP). AMH and inhibine were measured by ELISA (Immunotech AMH/MIS enzyme immunoassay). DHT and 17 hydroxypregnenolone were measured by chromatographic **se**paration on celite and radioimmunology with tritiated tracer.

### 3.2 Hormonal stimulation test

For the GnRH test: A bolus Stimu‐LH 50 given by intravenous injection (100 μg/m^2^) was used to test FSH and LH at time 0, 10′, 20′, 40′, 60′, and 120′.

For the hCG test: protocol study r‐HCG (Ovitrelle*) (Dr S. Cabrol and N. Lahlou, Hôpital Armand Trousseau Paris): Three subcutaneous r‐HCG injections (1625 IU) were performed at days 0, 3, and 6. AMH and inhibine B were assessed at day 0 and testosterone at days 0, 3, and 7.

### 3.3 Results: Hormonal status

The first hormonal basic investigations were conducted after 1 month of life (Table 1). Testosterone and AMH were slightly below standard, inhibine was at the lower limit of standard, and DHT and the testosterone/DHT ratio were normal. The amount of adrenal hormones was normal.

Further dynamic investigations past 10 months old consisted in a LHRH test and a r‐hCG test. Response to the LHRH test was too high, with a spike, particularly for FSH. At the age of 14 months, the child underwent a stimulation test with recombinant human gonadotropin.

Basic hormonal investigations were repeated at 6, 11.5, and 13.5 years.

As a result, the testis was considered as partially functional, but with a low response as well for the leydigian as for the sertolian functions.

## 4 CYTOGENETICS and MOLECULAR ANALYSIS

### 4.1 Ethical compliance

Genetic analyses were all performed after collecting a signed information form in compliance with French ethic laws.

### 4.2 Karyotype, FISH and genes

Chromosome analysis was performed with cultured blood lymphocytes, using RHG and GTG banding, according to standard cytogenetic techniques. The karyotype (300 bands) was 46,XY. Fluorescent in situ hybridization (FISH) studies were carried out on metaphase spreads, using X centromeric region probe, Y centromeric region probe, *SRY* (OMIM 480000) probe, and 22q11 probe (*TUPLE* 1; OMIM 600237) (Abbott). Whole subtelomeric regions of the chromosomes were also studied with specific probes from Abbott.

*High‐resolution banding* (550–850 bands), obtained after cell culture synchronization and 5‐bromo‐2′‐deoxyuridine (BrdU) incorporation.

*Following genes were sequenced:* the androgen receptor *(AR*; OMIM 313700) (exons 2–8), the 5 α‐reductase type 2 (*SRD5A2;* OMIM 607306; *5 exons*)*,* and the *WT1* (OMIM 607102; *10 exons*).

### 4.3 Results

#### 4.3.1 Initial investigation

A new karyotype analysis was performed on a blood sample shortly after birth and confirmed the genetic sex (46,XY). FISH analysis using specific probes of chromosomes X and Y (centromere X, centromere Y and *SRY*) showed no abnormality of both gonosomes.

The androgen receptor, the five α‐reductase type 2, and the *WT1* genes were sequenced, and their open reading frames showed no abnormality.

#### 4.3.2 Investigation at follow‐up

The clinical evolution motivated a new genetic examination and new blood tests for cytogenetic analysis. The genetic clinical examination pointed at some dysmorphic features. The child presented a sloping forehead with microcephaly (−4 *SD*). The nasal bridge was prominent. The ears were prominent with hypoplastic lobules. The mouth was small with a thin upper lip. A single palmar crease was noted on the right hand. He also had a nasal speech.

**Figure 2:**
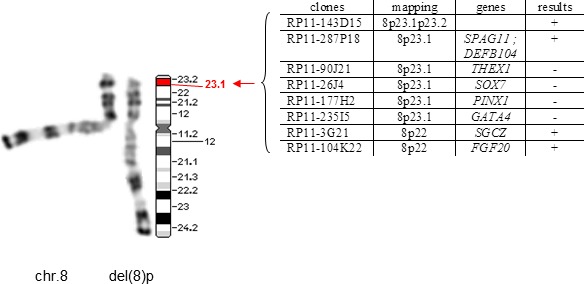
CGH array of chromosome 8

The standard karyotype (400 bands) was 46,XY. Analysis by FISH of the subtelomeric regions of the chromosomes revealed no abnormality. Chromosome investigations were completed by a high‐resolution analysis, which showed an interstitial deletion on the short arm of a chromosome 8. The final patient's karyotype could therefore be defined as 46,XY,del(8)(p23.1p23.1), revealing an interstitial deletion on the short arm of the chromosome 8. In order to characterize more precisely this deletion, we used a series of BAC encompassing the deleted region. The deleted region had an estimated length of 5.6 Mb and contained the genes*, PINX1 (OMIM* 606505)*, SOX7(OMIM* 612202), *and GATA4 (OMIM* 600576). (Figure 2).

The karyotype of the mother was normal. It was not possible to obtain the father's karyotype.

## 5 DISCUSSION

Table 2 summarizes the literature review we performed focused on the phenotype associated with 8p‐ deletion. Lubs and Lubs (1973) described the first case of a monosomy 8p. Since then, many cases of deletions of the short arm of chromosome 8, with different localizations and sizes, have been reported. We have identified 102 cases including 54 boys with 8p deletion: Among these, 63 had delayed psychomotor development or/and behavioral troubles, 57 had a heart malformation, 52 had other dysmorphic features, and 25 boys had a genitourinary anomaly.

**Table 2:** Cases affecting of 8p deletion; literature review and our case (^a^microcephaly, hypoplasia of corpus callosum, congenital diaphragmatic hernia)

Author (year)	Cases	Mental and/or behavior problems	Heart defect	Genitourinary anomalies	Dysmorphy and ^a^other frequent malformations	8p deletions
All publications before 1990	8M/6F	14	6	7	6	8p‐:**2**; del(8)(p21p23):**1**; del(8)(p21):**6**; del(8)(p22):**2**; del(8)(p11p21):**1**; del(8)(p23.1)**:2**
Blennow (1990)	1F	1	0	0	1	del(8)(p23.1):2
Pecile et al. (1990)	1M/1F	2	2	0	2	del(8)(p23.1); del(8)(p22)
Pettenati et al. (1992)	2M/1F	3	0	0	0	del(8)(p23.1)
Hutchinson et al. (1992)	2M/2F/1?	4	3	2(1UDT)	5	del(8)(p23.1)
Wu et al. (1996)	6M/8F	3	6	5	9	del(8)(p23.1)
Claeys et al. (1997)	3M/2F	5	3	2(UDT)	3	del(8)(p23.1)**:3**; del(8)(p22.1):**1**
Faivre et al. (1998)	1?	?	1	0	?	del(8)(p23.1)
Digilio et al. (1998)	3M/1F/3?	6	4	2 (hypospadias,UDT)	6	del(8)(p21):2;del(8)(p23.1):3
Pehlivan et al. (1999)	1M/4F	3	4	1	3	del(8)(p23.1p23.2):**2**; del(8)(p23.1):**3**
Devriendt et al. (1999)	3M	3	2	1	1	del(8)(p23.1)**:2**; del(8)(p12p22)**:1**
Reddy (1999)	3M/3F	2	2	0	2	del(8)(p23.1):**4;** del(8)(p23.1p23.1):**2**
Bhatia, Suri, Bundy, and Krauss (1999)	1M	–	1	0	1	del(8)(p23.1)
Giglio et al. (2000)	7M/5F	5	7	2 (hypospadias,UDT)	1	del(8)(p21.3):2; del(8)(21): 2; del(8)(p23.1):1;del(8)(p23.2):1 del(8)(p23.3):2
Gilmore, Cuskelly, Jobling, and Smith (2001)	1F	0	0	0	0	del(8)(p23.1)
Borys and Taxy (2004)	1F	0	1	0	0	del(8)(p23.1)
Shimokawa et al. (2005)	1M	0	1	1(UDT)	0	del(8) (p23.1 p23.1)
Sherr et al. (2005)	1M	1	1	0	1	del(8) (p23.1 p23.1)
López et al. (2006)	1M	0	1	0	1	del(8) (p23.1 p23.1)
Baynam, Goldblatt, and Walpole (2008)	1M	0	1	1(UDT)	1	del(8)(p23.1)
Páez et al. (2008)	2?	2	2	0	–	del(8)(p22p23.3); del(8)(p23.1p23.1)
Wat et al. (2009)	3M/1F	0	4	0	1	del(8) (p23.1 p23.1)
Ballarati et al. (2011)	1M	1	1	0	1	del(8) (p23.1 p23.1)
Willemsen et al. (2009)	1M/1F	2	1	1 (hypospadias)	2	del(8)(p21.3p12); del(8) (p21.2p12)
Chien et al. (2010)	1M	1	0	0	–	del(8)(p23.2)
Woo and Li (2011)	1M	1	0	0	1	del(8)(p23
Guimot et al. (2012)	1F/1M	1/?	2	–	–	del(8)p23.1p22/del(8)p23.1p22
Burnside et al. (2013)	2M/1F	3	0	0	3	del(8)(p23.3‐p23.2
Molck et al. (2015)	1F	1	1	–	1	del(8)p23.1
Our patient (2016)	1M	1	1	1(DSD)	1	del(8)(p23.1p23.1)
Total	102+1 cases 54+1 M/41 F/7?	64 (62.7%)+1	57 (55.9%)+1	25 (24.5%)+1 (46.3% M)	52 (50.9%)+1	

Notes

The numbers in bold indicate the number of a given mutation in the each sample.

Our patient presented some only of the phenotypical features reported in the literature (Claeys et al., 1997; Devriendt et al., 1999; Digilio, Marino, & Guccione, 1998; Faivre et al., 1998; Hutchinson, Wilson, & Voullaire, 1992; Pecile, Petroni, Fertz, & Filippi, 1990; Pehlivan et al., 1999; Pettenati et al., 1992; Sangeeta, Blennow, & Brondum‐Nielsen, 1990; Wu et al., 1996). The acquired microcephaly (Sangeeta et al., 1990), the abnormality of the genital organs for a boy (Digilio et al., 1998; Giglio et al., 2000), the delayed acquisition of language, and the hyperactive and impulsive behavior (Claeys et al., 1997) in a context of mild mental retardation (Fan, Siu, Jung, Farrell, & Cote, 2001; Fryns, Kleczkowska, Vogels, & Van den Berghe, 1989; Pettenati et al., 1992) are suggestive of such a chromosomal deletion. However, there was no initial congenital heart disease although cardiac malformations are described in 56% of the cases (Devriendt et al., 1999; Pehlivan et al., 1999; Watt, Battle, Li, & Duncan, 2004; Willemsen, Leeuw, Pfundt, Vries, & Kleefstra, 2009). Patients without cardiac problems are thought to have a smaller, more distal, deletion (5‐cM region, critical for cardiac development) (Giglio et al., 2000). In this particular case, our patient developed left ventricular noncompaction, a rare type of cardiomyopathy, that still could be associated to the chromosome 8 deletion, despite being atypical and only described once in the literature (Oechslin & Jenni, 2011). GATA4 mutations are known causes for cardiac septal defect but not for cardiomyopathy, even less ventricular noncompaction (Garg et al., 2003).

Not all of these children are born “small for gestational age”, and this child was harmonious at birth, at −1 *SD* for the three measurements (weight, height, cranial circumference). The postnatal growth is often, but not always, slowed down presently: Child's height and weight development was at +1 *SD*, with a microcephaly at −4 *SD* (Sangeeta et al., 1990).

The child's psychomotor and behavioral development is consistent with most described cases. The hypothesis has been formulated that a gene correlated with this type of behavior is located on the deleted distal zone of the short arm of chromosome 8 (Claeys et al., 1997).

The genital malformation was the first clinical feature to be diagnosed at birth in our case. We found well described by Digilio et al. (1998) both 46,XY DSD cases similar to ours, one with scrotal hypospadias and the other with hypospadias and bilateral cryptorchidism. In these cases, it was a de novo del (8)(p21), with larger deletions than in our case—del(8)(p23.1p23.1). These 8p23 deletions, particularly studied by Giglio et al. (2000), are often, but not always, associated with cardiac anomalies.

We wondered if any gene coding for sexual determinism was located in the deleted region. The *GATA4* and S*OX7* genes are located on the deleted zone of the short arm of chromosome 8. *GATA4* may regulate a set of cardiac‐specific genes, plays a crucial role in cardiogenesis, and is involved in the early development of the primary gonad. This gene acts as a secondary transcription factor of sexual determination. It is expressed in Sertoli cells during the fetal and postnatal period, with maximal expression between 19th and 22nd week of gestation, at a time when FSH secretion is high. *GATA4* is expressed in Leydig cells during fetal life and puberty, when active testosterone synthesis begins in the testis, suggesting it plays a role in androgen steroidogenesis (Katoh, 2002; Ketola et al., 1999, 2000; Lasala, Carre‐Eusebe, Picard, & Rey, 2004; LaVoie, 2003; Mazaud Guittot et al., 2007; Morel, Mallet, & Menassa, 2006; Oreal, Mazaud, Picard, Magre, & Carre‐Eusebe, 2002; Verloes, 1998; Viger, Mertineit, Trasler, & Nemer, 1998). In AMH‐positive cells of the spermatic cord (Sertoli cells), AMH is coexpressed with *DMRT1* (OMIM 602424), *SF1(OMIM* 601516), *WT1*,* GATA4,* and *SOX9* (OMIM 608160). *GATA4* is a regulating factor of AMH's transcription (Lasala et al., 2004; Oreal et al., 2002; Verloes, 1998). *SOX7* and *GATA4* are competitive activators of Fgf3 (*FGFR3*; OMIM 164950), *GATA4* being the primary activator and *SOX7* a minor activator in Fgf3 expression (Katoh, 2002). However, in animal experimentation, Watt et al. (2004) reported that mouse embryos displayed heart defects but no genital malformation was described.

With regard to the severe sexual development disorder observed in our case and in the five other boys with severe hypospadias, what could the function of the genes deleted in this microdeletion del(8)(p23.1p23.1) be? With 6 cases of severe DSD among 54 males with 8p deletion documented after 1990, the frequency of this type of disorder seems to be far higher (1.1%) than what was previously admitted (one case of severe hypospadias for 1,500 boys born alive, i.e., 0.7 for 1,000). There is not enough evidence to confirm, presently, that there is a direct relation between microdeletion 8p and 46,XY DSD. Further investigations are necessary, like determining the frequency of severe hypospadias associated with chromosomic anomalies.

## 6 CONCLUSION

In this case report, we aimed to stress the necessity of a multidisciplinary management, where medical, surgical, radiological, endocrinological, pathological, and genetic specialists collaborate. Without such a management, it would not have been possible to diagnose this deletion of the short arm of chromosome 8 affecting a boy with 46,XY DSD, microcephaly, particular morphotype, delayed acquisition, and behavioral disorders.

It is also worth to note that, even if the genes *GATA4* and *SOX7* are known for their role in sexual determination, this deletion has mainly been associated with cardiac abnormalities and slightly less often with genitourinary anomalies, but even more rarely with severe insufficient masculinization. The *GATA4* mutation is usually not found in hypospadias cases; so could it be a fortuitous association?

In conclusion, in the presence of a gonadal dysgenesis even of mild clinical expression, it proved to be useful, in collaboration with the clinical geneticists, to use cytogenetic methods to search for an anomaly on chromosome 8, where the p21 to p23 zone might be incriminated in male's sexual determinism. Further studies are needed to confirm this hypothesis.

## DISCLOSURE

The authors have no conflict of interest to declare.
